# Upper Gastrointestinal Disorders in Adult Patients with Intellectual and Developmental Disabilities

**DOI:** 10.7759/cureus.15384

**Published:** 2021-06-02

**Authors:** Jack S Curtis, Sara E Kennedy, Barrett Attarha, Linda Edwards, Rafik Jacob

**Affiliations:** 1 Internal Medicine, University of Florida College of Medicine, Gainesville, USA; 2 Internal Medicine, University of Florida College of Medicine – Jacksonville, Jacksonville, USA

**Keywords:** dysphagia, aspiration pneumonia, dental caries, intellectual and developmental disabilities, gerd, gastric emptying disorders

## Abstract

The purpose of this literature review is to address the diagnosis and treatment of upper gastrointestinal (GI) disorders in patients with intellectual and developmental disabilities (IDD). Manifestations of upper GI dysmotility and disorders include dysphagia, pulmonary aspiration, malnutrition, gastroesophageal reflux, and gastritis, all of which can impact a person’s quality of life and lead to chronic, life-threatening conditions. This article will explore the existing diagnostic methods and treatments for gastrointestinal disorders as they relate to patients with IDD.

## Introduction and background

IDD is characterized by significant limitations both in intellectual functioning and in adaptive behavior as expressed in conceptual, social, and practical adaptive skills. This disability originates during the developmental period, which is defined operationally as before the individual attains age 22 [1]. IDD can begin at any point during a person’s developmental period, most often before a person is born, and generally lasts for the duration of that person’s life. Etiologies of developmental disabilities include a mix of factors such as complications at birth, prenatal infections, exposure of the child and/or mother to environmental toxins, maternal health and prenatal behaviors, and genetics [2].

Dysmotility of the upper gastrointestinal tract represents a severe constellation of symptoms resulting from malfunction of the stomach, small intestine, or large intestine [3]. Concerns with upper gastrointestinal dysmotility are relevant to patients with IDD as there can be unique challenges in the diagnosis and medical care within this patient population. Chronic upper gastrointestinal (UGI) dysmotility problems for patients with IDD include dysphagia (60%), pulmonary aspiration (41%), malnutrition (33%), gastroesophageal reflux (GERD) (32%), and gastritis (32%). Uncoordinated swallowing can lead to inadequate nutrition and caloric intake, in addition to impacting a person’s quality of life [4]. Repeated pulmonary aspirations, 80% of which are silent, can lead to chronic lung disease [5]. Chronic GERD can lead to Barrett’s esophagus and ultimately carcinoma of the esophagus [4]. Infection with Helicobacter Pylori (H. Pylori) is higher in children with developmental disabilities who live in group homes than in the general population, and H. pylori can lead to peptic ulcer disease and potentially gastric adenocarcinoma [5].

## Review

Diagnosis

In patients with IDD, the history may be vague or even misleading, and is often provided by a surrogate. This often leads to a reliance on diagnostic tests. A variety of testing modalities are used to diagnose motility disorders of the UGI. In the assessment of dysphagia and chronic aspiration, a barium swallow or a modified barium swallow study should be performed to further elucidate the cause. This is preferable to invasive testing such as upper endoscopy, which can have an increased risk if the patient is not fully cooperative. In a barium swallow study, repeated x-rays are taken as the patient swallows barium, and in a modified barium swallow study, video fluoroscopy is taken as the patient swallows barium. Both studies provide insight into the structure and function of the patient’s esophagus and determine if aspiration is occurring. GERD and its sequelae, such as Barrett’s esophagus, as well as gastritis can be further assessed using upper endoscopy when clinically indicated [6]. Upper endoscopy should be considered in patients who have symptoms suggestive of complicated disease (eg, dysphagia, unintentional weight loss, hematemesis), and failure to respond to appropriate antisecretory medical therapy. Diagnosis of H. pylori infection in adults with IDD can present challenges since the gold standard of testing, the urease breath test, can be difficult to perform in those with lower cognitive function. Stool antigen and blood antibody testing are the standard in this population [7]. Nutritional status can be evaluated non-invasively with body mass index, skinfold thickness measurements, and bioelectrical impedance analysis, which uses the conduction of electrical currents to determine the percentage of fat, water, and muscle present [8].

Dysphagia

Oropharyngeal dysphagia is the difficulty with or abnormal transfer of food or liquids from the oral cavity through the pharynx and into the esophagus. Prior studies among the pediatric population have identified that developmental abnormalities such as craniofacial abnormalities or faulty sensory and motor development predispose patients to dysphagia [9]. The more severe forms of IDD tend to be more commonly associated with dysphagia. While the exact prevalence has proven to be difficult to ascertain, prior studies have estimated the prevalence of dysphagia to be between 8.1% to 11.5% in patients with IDD [10]. However, many studies have also demonstrated that dysphagia is often under-reported, and when observed by certified speech therapists in smaller studies, the prevalence has been greater than 50% [11-12]. Dysphagia itself may lead to serious health complications and both psychological and social suffering [13]. Dysphagia increases risk for aspiration pneumonia, choking, and decreased hydration and nutrition status [13]. Traditionally, once oropharyngeal dysphagia is excluded, esophageal dysphagia is evaluated via visualization of the esophagus either by endoscopy or contrast radiography, and then the functional status of the esophagus is evaluated by high resolution manometry. This can be challenging in patients with IDD who might not tolerate the placement of a nasoesophageal probe. Newer technologies such as EndoFLIP might present an alternative mode of testing, as the function can be assessed while a patient is sedated using a transoral catheter with balloon to simulate the presence of a food bolus and then assess the esophageal response by measuring pressure and impedance [14].

Gastroesophageal Reflux Disease

Gastroesophageal reflux disease occurs when stomach contents reflux up into the esophagus and cause complications and/or symptoms such as burning, chest pain, and dysphagia [15-16]. It is believed that neuromuscular dysfunction is the primary etiology as to the increased prevalence in patients with IDD [17]. The prevalence of GERD in patients with IDD has been found to be approximately 50% [18], whereas the prevalence of GERD in the general population is approximately 10% to 20% in Western countries and less than 5% in Asia [19]. Serious complications from GERD may arise if left untreated, such as erosive esophagitis, Barrett’s esophagus, and esophageal stricture. For the clinician, it is important to consider rumination syndrome which is common in patients with IDD. Here, patients may regurgitate food and then re-swallow or expel it. A careful history may indicate this condition, which is associated with weight loss, malnutrition, dental problems, halitosis, and electrolyte disturbance [20].

Aspiration Pneumonia

Aspiration pneumonia is one of the more serious sequelae of upper gastrointestinal dysmotility and is typically secondary to gastroesophageal reflux or dysphagia. It is believed to be the leading cause of mortality in patients with dysphagia secondary to neurologic dysfunction (300,000-600,000 deaths annually in the United States) [21]. Aspiration pneumonia occurs when gastric or oropharyngeal contents enter the lower pulmonary system, bringing with it enteric organisms and potentially harmful exogenous substances [22]. These bacteria and gastrointestinal contents can lead to inflammation and infection of the lungs. Patients with neurologic deficits and mechanical abnormalities, such as patients with cerebral palsy, are particularly at increased risk for aspiration. While the incidence and prevalence of aspiration pneumonia in the general community is difficult to define [23], in “one study of patients with nursing home-acquired pneumonia and controls with community-acquired pneumonia, the incidence of aspiration pneumonia was 18 percent and 5 percent, respectively” [24]. The increased risk of aspiration pneumonia in patients with IDD is multifactorial, and as discussed previously, dysphagia and reflux are two of the primary risk factors, along with dependence for feeding and oral care [25]. Poor dental hygiene also increases risk of aspiration pneumonia, which is thought to occur due to potentially more pathogenic oral bacteria and higher quantity of bacteria [26-27]. When there is clinical suspicion of aspiration, a videofluoroscopic swallow study should be performed to aid in diagnosis [28].

Dental Caries

While often overlooked as a sequela of upper gastrointestinal dysmotility, dental caries poses a serious health concern as a consequence of both poor oral hygiene and gastroesophageal reflux [29-30]. A longitudinal 8.5 year study that followed 124 individuals ages 21-40 with IDD demonstrated that while the overall incidence of caries was low (0.51 new lesions per year), patients with poor ability to cooperate and more severe disabilities had an increased risk for impaired oral health [31]. Other studies in patients with cerebral palsy have also found that “the more severe the neurological damage, the more frequent is the presence of the biting reflex and consequently, the higher is the risk of oral diseases in this population due to the difficulty to perform an adequate oral hygiene” [32]. Similarly, among patients with cerebral palsy, those who had IDD had higher rates of caries when compared to those without intellectual disability [33]. Oral care should take place regularly in patients with IDD and can usually be accomplished in a general dental practice.

Gastric Emptying Disorders

In the absence of a structural change to the stomach, disorders related to proper emptying of gastric contents into the duodenum occur secondary to neurogenic or myogenic disorders, psychological stress, and/or neuronal developmental disorders. Most commonly, delayed gastric emptying is seen in those with reflux esophagitis, dyspepsia, autonomic neuropathies, psychiatric disorders, and conditions like systemic sclerosis [34]. While the exact prevalence of delayed gastric emptying in those with IDD has not been ascertained, a study of 58 children with cerebral palsy found that 67% of participants had delayed gastric emptying as demonstrated by gastric scintigraphy [35]. The prevalence of delayed gastric emptying or gastroparesis, in the general population is 0.16% [36]. Sequelae of delayed gastric emptying include malnutrition, reflux esophagitis, and constipation, which is a disorder of the lower gastrointestinal tract [35].

Eosinophilic Esophagitis

Esophagitis is typically caused by reflux of stomach contents, medication irritation, infections, or allergies. Studies have shown that patients with IDD are also at greater risk of eosinophilic esophagitis in addition to gastroesophageal reflux. Diagnostic criteria for eosinophilic esophagitis include “symptoms of esophageal dysfunction and at least 15 eosinophils per high-power field on esophageal biopsy and after a comprehensive assessment of non-EoE disorders that could cause or potentially contribute to esophageal eosinophilia” [34]. In a retrospective study of 229 children diagnosed with eosinophilic esophagitis, 29 (12.7%) children were found to have autism spectrum disorder and 70 (30.6%) had developmental disorders [35]. This is a significant difference from the national prevalence of autism spectrum disorder (0.5%-3%) and developmental disorders (17.8%) [35]. While this may be secondary to increased access to and contact time with medical professionals within this patient population, it has been hypothesized that there is a shared immunologic pathway between these conditions [35]. Multiple studies have found increased levels of Th2 cell activation and its related cytokines, which are associated with eosinophilic esophagitis in patients with autism spectrum disorder [36-37]. Because peripheral eosinophilia is not necessarily related to EoE, endoscopy with biopsy of the lower and mid esophagus is required to make the diagnosis.

Treatment Options

Diet Modifications

In a 2008 study conducted with 249 children and 158 adults with IDD and concurrent gastrointestinal, swallowing, or nutritional problems, it was found that conservative management was the most successful intervention for dysphagia, aspiration, and malnutrition in most participants [5]. It was found that the study participants who had their diets modified with soft foods had the lowest occurrence of complications of dysphagia and were followed closely for appropriate weight gain and symptom recurrence. Of the 294 children enrolled in the modified diet, 31% (91 total) required a gastrostomy tube placement because of failure of weight gain or severe dysphagia, whereas 4.4% (7 total) of the 158 enrolled adults required a gastrostomy tube placement [5]. Fundoplication for refractory gastroesophageal reflux was performed in 25% (74 out of 294) of children participants and 1.3% of adult participants (2 out of 158) [5].

G-tubes/J-tubes

If utilizing diet modifications is impractical or unsuccessful, such as in patients with extremely severe oropharyngeal dysphagia, using a gastrostomy or jejunostomy tube may be practical. This provides the benefit of delivering adequate amounts of nutrition to patients while potentially decreasing aspiration risk. In patients with no history of aspiration pneumonia, prophylactic use of these feeding tubes remains controversial, and some providers may feel uncomfortable prescribing this therapy. However, a recent retrospective study of 4,112 children aged 0-18 found that gastrostomy tube insertions increased by 140% between 1998 and 2014, and that they are being utilized at earlier ages [38]. A more recent 18-month cross-sectional study conducted in 2019 among 474 patients with severe and complex disability found that the most common medical device used in aging patients with these illnesses was gastrostomy tubes. This study identified that 35.6% of patients aged 18-34, 21.2% of patients aged 35-49, and 21.2% of patients age 50-68 utilized gastrostomy devices, primarily prescribed as a means of reducing risk of aspiration pneumonia and alleviate symptoms of GERD [39]. Another study showed that serum levels of folate and vitamin B6 are significantly higher in patients with severe motor or IDD using enteral feeding when compared to oral feeds. This study also demonstrated that among patients using enteral feeds, levels of B12 are significantly higher when using a gastrostomy tube compared to a jejunostomy tube [40]. However, gastrostomy tubes have been demonstrated to be inferior to jejunostomy tubes in prevention of aspiration pneumonia due to less likelihood of displacement or clogging [41]. In addition, following proper feeding protocols, such as keeping the head of the bed elevated at a 30-degree angle can also help reduce the risk of aspiration when utilizing tube feeds [42]. Involving speech-language pathologists in the care of patients who may benefit from feeding tubes is extremely helpful. These medical professionals are able to provide both evaluations of the severity of dysphagia and instruction on proper feeding tube use. This has proven to be helpful in other patient populations with feeding difficulties such as patients with advanced dementia [43].

Antacids, Proton Pump Inhibitors, and Histamine-2 Receptor Antagonists

Some of the most widely used medical treatments in patients with gastroesophageal reflux disease, not only in patients with IDD but also in the general population, are antacids, proton pump inhibitors, and H2 blockers. Antacids are comprised of compounds such as magnesium trisilicate, aluminum hydroxide, or calcium carbonate. These act by neutralizing acid in the stomach, thereby reducing symptoms of heartburn. These typically do not cause significant side effects, but in compounds containing magnesium, diarrhea may occur which may interfere with absorption of other medication. Hypermagnesemia and hypercalcemia should also be considered in patients with significant renal disease. Proton pump inhibitors (PPIs) work by irreversibly binding and inhibiting the hydrogen-potassium-ATPase pump in the stomach, thereby blocking acid secretion. PPIs are the mainstay treatment for GERD and peptic ulcer disease. However, chronic treatment with PPIs has shown an increased risk of C. difficile infection, diarrhea, hypomagnesemia, vitamin B12 malabsorption, decreased calcium absorption, acute interstitial nephritis, and chronic kidney disease. For these reasons, magnesium and B12 levels should be monitored in patients on chronic PPIs. Lastly, Histamine-2 antagonists block H2 receptors on gastric parietal cells. While adverse effects are rare, inhibition of the hepatic cytochrome P450 system can lead to drug interactions with medications that are hepatically excreted. While few studies have been conducted to assess the efficacy of these treatment options specifically in patients with developmental or intellectual disability, these mainstay treatments for GERD and peptic ulcer disease are appropriate treatments in these patient populations. However, a 2018 study of 677 adults with intellectual disability aged 40 and above in Ireland found that 27.9% of their participants were on chronic PPIs. Of those participants on PPIs, only 43.9% had an indication for PPI use (GERD, peptic ulcer or/and NSAID use) [44]. Chronic use warrants consideration of drug-drug interactions and electrolyte imbalances and should only be considered in patients with clear indications for their use.

## Conclusions

The diagnosis and treatment of dysmotility of the upper gastrointestinal (UGI) tract in patients with IDD presents unique challenges as a result of impairments in behavior, language, learning, and/or physical growth. While there are special concerns in the care of this patient population, there are effective diagnostic tools and treatments available, which may vary from those used in the general population. Practitioners should be aware of the specialized protocols available to provide optimized care. Lastly, it should be recognized that many studies referenced in this article were done in pediatric populations, but the data can still be extrapolated to adults in whom we do not have a substantial number of studies.
